# Demonstration of a Chemical Recycling Concept for Polybutylene Succinate Containing Waste Substrates via Coupled Enzymatic/Electrochemical Processes

**DOI:** 10.1002/cssc.202402515

**Published:** 2025-02-19

**Authors:** Richard Buchinger, Sabrina Bischof, Ole Nickel, Vanessa Grassi, Jasmin Antony, Markus Ostermann, Soniya Gahlawat, Markus Valtiner, Robert Meißner, Georg Gübitz, Christian M. Pichler

**Affiliations:** ^1^ Institute of Applied Physics Vienna University of Technology 1040 Vienna Austria; ^2^ Institute of Environmental Biotechnology University of Natural Resources and Life Sciences 3430 Tulln Vienna Austria; ^3^ Institute for Modeling of Soft Matter Hamburg University of Technology 21073 Hamburg Germany; ^4^ Institute of Surface Science Helmholtz-Zentrum Hereon 21502 Geesthacht Germany; ^5^ Center for Electrochemical and Surface Technology 2700 Wr. Neustadt Austria

**Keywords:** Enzymatic hydrolysis, Electrocatalytic succinic acid decarboxylation, Ethene production, Plastic waste recycling

## Abstract

Chemical recycling of polymer waste is a promising strategy to reduce the dependency of chemical industry on fossil resources and reduce the increasing quantities of plastic waste. A common challenge in chemical recycling processes is the costly downstream separation of reaction products. For polybutylene succinate (PBS) no effective recycling concept has been implemented so far. In this work we demonstrate a promising recycling concept for PBS, avoiding costly purification steps. We developed a sequential process, coupling enzymatic hydrolysis of PBS with an electrochemical reaction step. The enzymatic step efficiently hydrolyses PBS in its monomers, succinic acid and 1,4‐butanediol. The electrochemical step converts succinic acid into ethene as final product. Ethene is easily separated from the reaction solution as gaseous product, together with hydrogen as secondary product, while 1,4‐butanediol remains in the aqueous solution. Both reaction steps operate in aqueous solvent and benign reaction conditions. Furthermore, the influence of electrolyte components on the electrochemical step was unraveled by applying molecular dynamic simulations. The final coupled process achieves a total ethene productivity of 91 μmol/cm^2^ over a duration of 8 hours, with 1110 μmol/cm^2^ hydrogen and 77 % regained 1,4‐butanediol as valuable secondary products.

## Graphical abstract

Solutions to convert ever increasing amounts of plastic waste are urgently needed. A bio‐electrocatalytic concept for Polybutylene succinate recycling was proposed yielding ethene, 1,4‐butanediol and H_2_ as value‐added products. Individual optimization of bio‐ and electrochemical process steps revealed different requirements on the individual reaction media. Nevertheless, successful coupling of both steps was achieved by optimization of the reaction media.

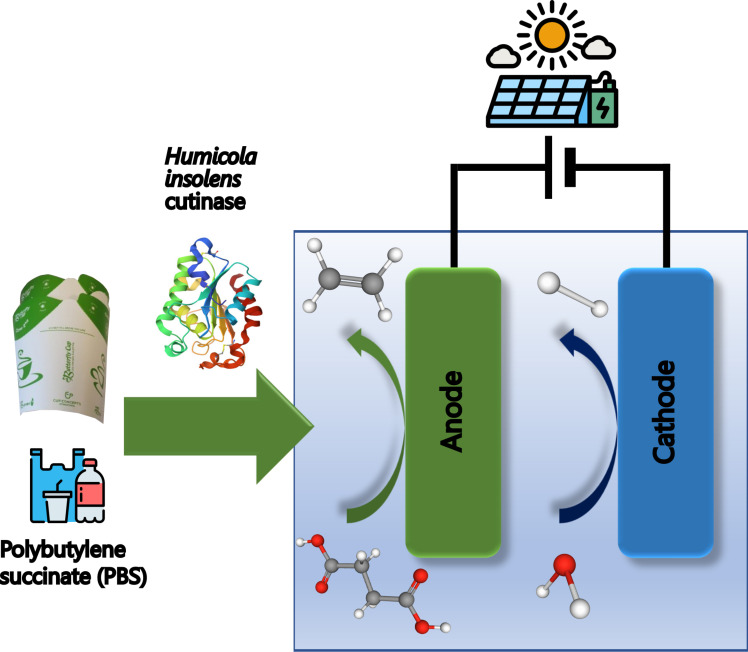

## Introduction

To enable genuine circular economy, available waste streams must be converted into industrially useful value‐added products and reintroduced into the economic cycle. For plastic waste streams, 79 % are still not recycled but disposed in landfills or incinerated.^[1]^ Electrochemical conversion of plastic waste into defined chemical compounds has been demonstrated to be a viable and sustainable pathway, to generate value‐added products for industrial utilization.^[2]^ Nevertheless, most electrochemical pathways focus on the conversion of polyethylene terephthalate (PET), which is already the polymer material with the highest recycling quota, while other polymer materials are only rarely considered.^[3]^ In most cases PET is enzymatically^[4]^ or chemically hydrolysed in its monomers, terephthalic acid and ethylene glycol, and only ethylene glycol is converted in anodic reactions, yielding oxygenated products such as glycolic acid or formic acid.[^5^, ^6^, ^7^, ^8^, ^9^, ^10^] The isolation and purification of such oxygenated products from aqueous solutions is complex and costly.^[11]^

Despite the progress in this field, only a few examples for the electrochemical conversion of alternative polymers or the formation of other products (not oxidized derivatives of ethylene glycol) are reported. One example is the two‐step conversion of polyethylene (PE), in which PE is chemically converted into carboxylic acids, which react in a second, electrochemical step to form valuable alkene products, such as ethene and propene.^[12]^ Ethene is a crucial platform chemical for PE production and further organic intermediates (e. g. ethylene oxide or vinylchloride).^[13]^ The vast share of commercially produced ethene is still made from fossil fuels via steam‐cracking processes contributing to the high CO_2_ emissions in chemical industry.^[14]^ Therefore, the anodic electrochemical ethene formation from carboxylic acids is a highly attractive reaction, as the necessary electricity can be obtained from renewable sources, reducing the CO_2_ footprint significantly.^[15]^ Additionally, carboxylic acid substrates can be derived from several waste streams (plastic, biomass, food waste), enabling a truly sustainable and circular pathway for ethene production.

The main challenge for the electrochemical conversion of waste streams is to make the insoluble waste substrates accessible for electrochemical conversion in aqueous electrolytes. While chemical processes such as alkaline hydrolysis of PET or oxidative decomposition of PE with HNO_3_ have proven to be effective, enzymatic processes would be preferable, as they do not require the use of additional chemicals, are performed under milder conditions and allow stepwise recovery of building blocks from complex materials.^[12]^ Furthermore, enzymes represent a powerful tool to specifically recover monomers from blended materials, multilayers or mixed plastic waste.^[16]^

Recently, the enzymatic hydrolysis of PET, coupled with selective, photoelectrochemical conversion of the obtained ethylene glycol was demonstrated, increasing the sustainability of the overall process.^[17]^ Nevertheless, examples for direct coupling of enzymatic and electrochemical processes are rare, due to the complexity of this task. The various auxiliary chemicals (buffer, nutrients etc.) required for enzymatic reactions can interfere with electrochemistry. Therefore, often only mock substrates or highly purified substrates are used in many waste conversion approaches. Detailed understanding of the influence of enzymatic media onto electrochemical steps is essential to advance waste conversion processes, and reduce purification/separation steps, to increase the feasibility of those concepts.

In this study, sequential chemical recycling of polybutylene succinate (PBS), into various value‐added compounds was demonstrated for the first time, while minimizing the necessary purification and separation efforts. PBS is a polymer of great interest, with a production of 100 000 tons per year, as PBS is used as coating material for paper cups.^[18]^ There are no established recycling protocols for PBS in place especially when present in multilayer or blended materials for food packaging, tableware or mulch films. Hence, it is an ideal substrate for bio‐electrochemical conversion into value‐added products.^[19]^

**Figure 1:**
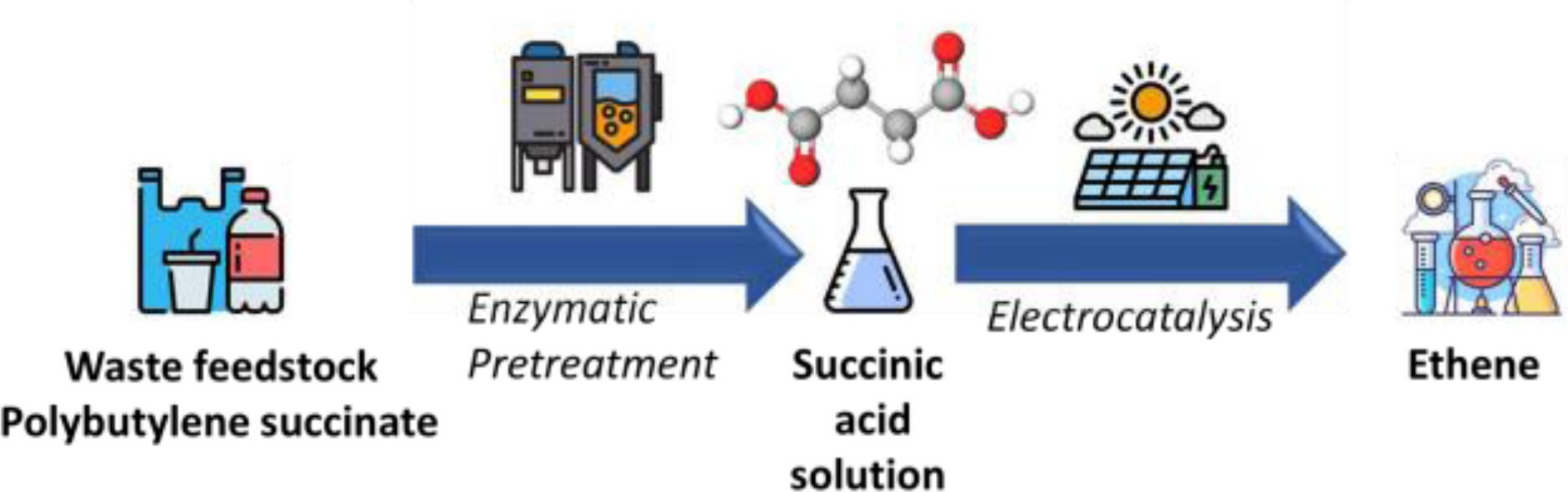
Scheme of the 2‐step process from PBS via the intermediate succinic acid to ethene using electrocatalysis.

We optimized the enzymatic hydrolysis step to efficiently convert PBS into its constituent monomers succinic acid (SA) and 1,4‐butanediol (BD), enabling full polymer hydrolysis. In a second electrochemical step the succinic acid is anodically converted into ethene (Figure 1) with concurrent formation of hydrogen on the cathode. As ethene is a gaseous product, released spontaneously from the reaction solution, no costly purification or isolation of products is necessary. BD as second hydrolysis product of PBS, remains in the aqueous solution. Alongside ethene, CO_2_ is formed during decarboxylation. When implementing this process, CO_2_ must not be released into the atmosphere. The difference in boiling points of the main reaction compounds (CO_2_: −78 °C, ethene: −104 °C, N_2_: −196 °C), would allow easy separation by cryo‐distillation and therefore efficient separation of CO_2_, which can be used in other processes or sequestered.[^20^, ^21^] Additionally, direct conversion concepts for CO_2_ from decarboxylations have been suggested, such as the conversion into styrene carbonate.^[22]^

In contrast to metals (which often favor Kolbe reaction), carbon‐based materials are known to favor the ethene formation pathway via intramolecular radical rearrangement.[^23^, ^24^] Thus, carbon felt was chosen as material for the investigations in this study (Pt, Ni or Au did not yield any ethene in screening experiments). Individual optimization experiments unveiled different requirements on the reaction media for the enzymatic and electrochemical step, making the efficient process combination a major challenge. However, by tuning the enzymatic hydrolysis conditions, it was possible to avoid any intermediate purification steps and directly utilize the hydrolysis solution for electrochemistry. Furthermore, the influence of buffers and electrolytes onto the electrochemical ethene formation could be determined by molecular dynamic (MD) simulations, allowing deeper insights into the electrochemical reaction mechanisms. The efficient combination of both steps to directly convert PBS into value‐added ethene, BD and hydrogen was demonstrated, extending the scope of bio‐electrochemical processes towards novel waste substrates and value‐added products, without the requirement of costly product purification processes.

## Results and Discussion

### Enzymatic Hydrolysis and Product Quantification

**Figure 2:**
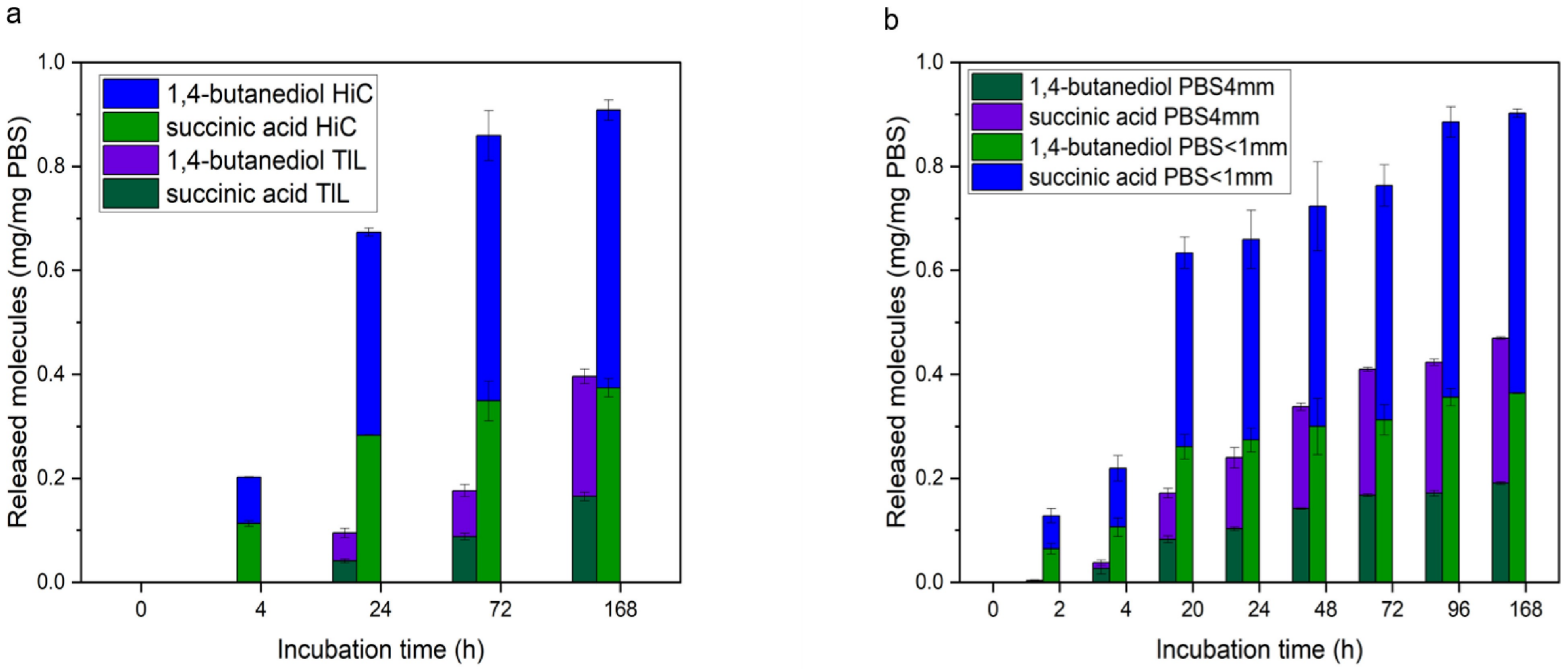
a) Comparison of enzymatic hydrolysis of PBS by the HiC cutinase and the TiL lipase using 10 U enzyme per mg of PBS. The release of monomers was monitored by using HPLC; b) Enzymatic degradation of PBS via HiC – comparison of product accumulation for PBS beads (4 mm) and powder (<1 mm) over 7 days.

In a first step, enzymatic hydrolysis of PBS by two enzymes, namely *Humicola insolens* cutinase (HiC) and *Thermomyces lanuginosus* lipase (TlL) was compared. HiC has been described to hydrolyse aromatic‐aliphatic polyesters including PET[^25^, ^26^] while hydrolysis of aliphatic polymers by *T. lanuginosus*, a well‐known lipase producer, was reported.^[27]^ After 7 d of incubation the polymers were completely decomposed when using HiC and 0.53 mg/mg PBS of succinic acid & 0.37 mg/mg PBS of 1,4‐butanediol were determined, whereas 0.23 mg/mg PBS of succinic acid & 0.17 mg/mg PBS of 1,4‐butanediol were obtained after incubation with TlL (Figure 2a). This means about 90 % of the initial mass has been converted into monomers during HiC treatments. Regarding the TlL experiment, about 40 % PBS source could be hydrolysed. Since at the identical enzyme activity of 10 U enzyme/mg PBS, a higher amount of monomers was recovered for HiC, this enzyme was used in all further experiments.

In the next step, the influence of the PBS particle size on enzymatic hydrolysis was studied. Clearly, a lower particle size was beneficial for hydrolysis (Figure 2b). For 4 mm beads 0.28 mg/mg PBS of succinic acid and 0.19 mg/mg PBS 1,4‐butanediol were released while beads with a size <1 mm were hydrolysed considerably faster resulting in 0.54 mg/mg PBS of succinic acid and 0.36 mg/mg PBS 1,4‐butanediol after 168 hours of incubation. In agreement with these findings, more efficient enzymatic hydrolysis for smaller particles has been reported for poly(ethylene 2,5‐furanoate).^[28]^

### Analysis of PBS Films

**Figure 3:**
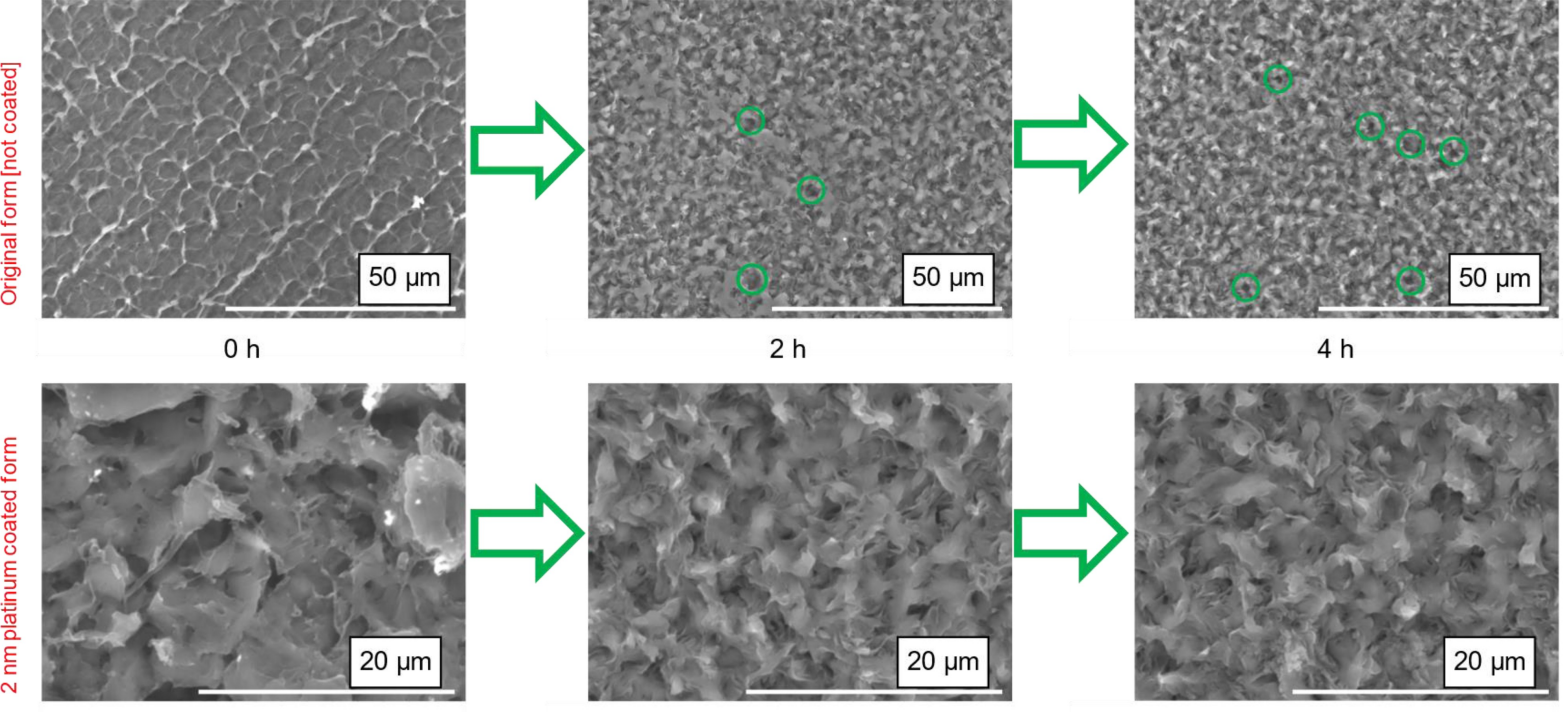
Surface morphology of a PBS film: non‐degraded, after 2 h incubation and after 4 h incubation with HiC in original imaging form (not coated) and 2 nm platinum coated form; the green circles indicate pores.

Hydrolysis of PBS‐films was further studied using FT‐IR. PBS‐films were retrieved partially intact after incubation and weighed, for assessing weight loss upon drying. FT‐IR measurements were carried out on PBS films to assess the peak changes (Figure S1) confirming the hydrolysis of the ester bonds, because of the hydrolytic activity of the enzymes, a detailed discussion is found in the SI. Figure 3 compares the surface morphology of untreated (blank) and hydrolysed PBS films. In the blank sample the surface is smoother and more regular, compared to the samples that have been incubated with the cutinase. In the untreated sample, a regular pattern of oblique lines (original, not coated form) can be observed: this is possibly an intrinsic property of the plastic sample due to manufacturing. Electron microscopy images of samples incubated over 2 h and 4 h in an enzyme solution containing 5 μM HiC illustrate that the number of pores increased with degradation time, and that the surface of the sample incubated over 2 h looks overall smoother than that of the sample incubated over 4 h. Obviously upon enzymatic hydrolysis, surface erosion takes place which increased the surface roughness.

**Figure 4:**
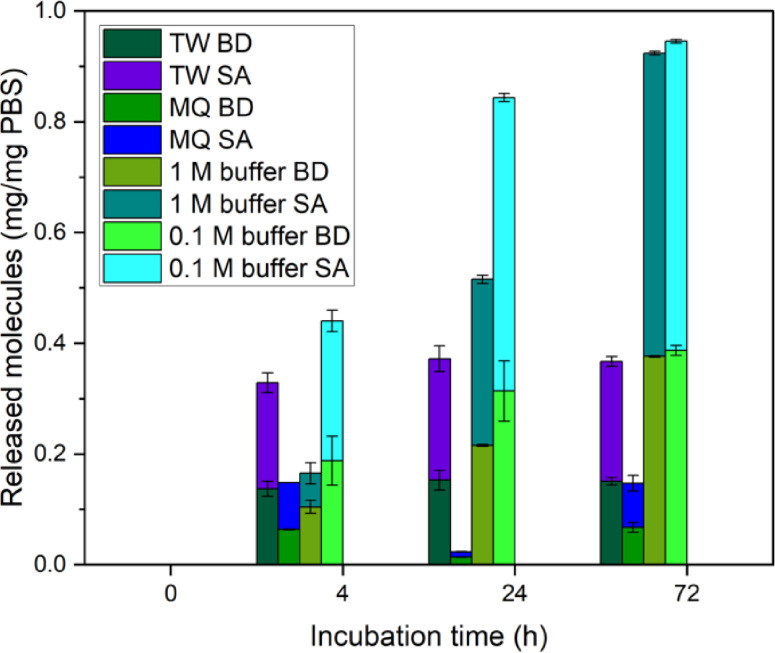
Enzymatic hydrolysis of PBS by HiC – product formation upon hydrolysis mediated by 4 U HiC/mg PBS in different media, Abbreviations: TW: Tap water, MQ: Milli‐Q water, SA: succinic acid, BD: 1,4‐butanediol, buffer: Potassium phosphate buffer.

The influence of different reaction media on the hydrolysis of PBS mediated by 4 U HiC/mg PBS over 3 days is shown in Figure 4.

Besides two different concentrations of potassium phosphate buffer (0.1 M and 1 M), tap water (TW) and Milli‐Q water (MQ) are compared. Within the first 24 h 0.1 M buffer led to faster hydrolysis than in 1 M buffer, while after 72 h the total amount of products was similar in both buffers, exhibiting close to full conversion of the initial PBS. In contrast, tap water, as well as Milli‐Q water conditions showed overall low hydrolysis yield. Expectedly, the pH immediately dropped from 7.4 (TW) and 6.7 (MQ) to 3.6 (TW) and 3.1 (MQ) due to the release of succinic acid strongly deactivating the HiC enzyme, causing low product yields (Figure S2). This illustrates the necessity of utilizing a buffer. However, there is no significant difference concerning the hydrolysis activity between the 1 M and 0.1 M buffer.

### Recovery of Building Blocks from PBS/Paper Cups

Due to their high specificity, enzymes offer potential in recovery of building blocks from the individual components in multilayer materials or from mixed wastes.^[29]^ This is highly relevant for PBS, as this polymer is mostly used as coating in paper cups. Hence, we have expanded our method to commercial PBS coated paper cups. Subjecting 2 g of shredded paper cups to enzymatic hydrolysis for 24 hours in 40 mL 0.1 M phosphate buffer, 2.32 mg/mL 1,4‐butanediol and 3.42 mg/mL succinic acid were released. Longer hydrolysis times did not increase the yield of monomers, indicating that all accessible PBS has been hydrolysed (Table S1). The observed weight loss is – with 17.8 % – higher than the expected 9.5 %, from the detected monomer concentrations. This could be due to the fact that cellulose fibrils entrapped by the PBS coating are released as well into the solution during PBS hydrolysis, additionally contributing to the mass loss.

After demonstrating the hydrolysis of PBS successfully also on real waste cups, the electrochemical step, for further converting the obtained succinic acid was optimized.

### Electrochemical Optimization

**Figure 5:**
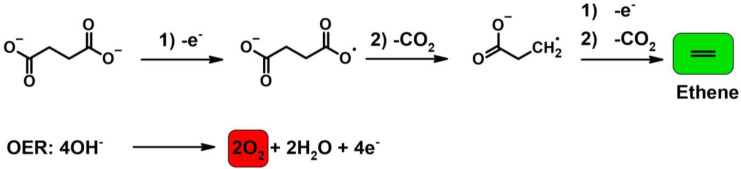
Desired reaction pathway starting from succinic acid forming the ethene and chemical equation of parasitic OER.

Before testing the solutions obtained from enzymatic hydrolysis, the electrochemical reaction was optimized by using standard solutions, containing 0.05 mol/L succinic acid. As electrolyte potassium phosphate buffer was used, to mimic the enzymatic hydrolysis solution. Thereby, the influence of phosphate concentration on the succinic acid to ethene conversion could be tested. Figure 5 shows the desired reaction pathway towards ethene and the unavoidable parasitic oxygen evolution reaction (OER). A blank test in pure potassium phosphate buffer without the presence of succinic acid, did not yield any ethene as product. Hence, it can be assumed that all ethene detected in the following experiments is derived from the succinic acid conversion, as demonstrated previously.^[15]^ It must be emphasized again, that the succinic acid to ethene conversion, is an anodic reaction. For all electrochemical tests, hydrogen evolution reaction (HER) is the balancing cathodic reaction, yielding H_2_ as valuable secondary product.

### Temperature and Voltage

**Figure 6:**
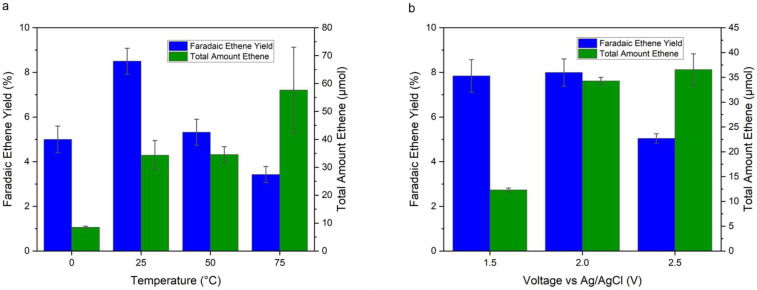
a) Faradaic ethene yields and total amount of ethene produced of electrochemical conversion of succinic acid to ethene for 0, 25, 50, 75 °C; 0.05 mol/L succinic acid, 0.5 mol/L phosphate buffer at pH=6, CA was conducted for 15 min at 2.0 V vs. Ag/AgCl (3 M KCl); b) Faradaic ethene yields at 25 °C and total amount of ethene produced by electrochemical conversion of succinic acid to ethene for voltages in CA of 1.5, 2.0 and 2.5 V vs Ag/AgCl (3 M KCl); 0.05 mol/L succinic acid, 0.5 mol/L phosphate buffer at pH=6, CA was conducted for 15 min.

Besides room temperature (25 °C), 3 more temperatures were tested (0, 50 and 75 °C) to check whether there is any dependence. However, neither lower, nor higher temperatures did improve the FY (Figure 6a). The total amount of ethene produced on the other hand was observed to increase with higher temperature. This can be explained by reduced solubility of ethene and acceleration of reaction rates at higher temperature. At 75 °C decomposition of the carbon felt electrode is observed, hence higher temperatures were not investigated.

Besides temperature, the applied voltage was screened. (Figure 6b) Three different voltages were tested to assess the dependence of the FY on the applied potential. Voltages of 1.5 and 2.0 V vs. Ag/AgCl (3 M KCl) result in 7.8 % and 8.0 % of FY respectively. Increasing the voltage to 2.5 V, led to a decrease of the FY to 5.0 %. As established in a previous study, the succinic acid to ethene conversion proceeds in parallel with the OER. Decreased Faradaic ethene yield is therefore explainable by increasing prevalence of OER.^[15]^ However, also side reactions (overoxidation of succinic acid and others) must be considered especially at high potentials of 2.5 V.

Based on these results, it was decided to conduct all following experiments at 25 °C and apply a potential of 2 V vs. Ag/AgCl (3 M KCl), as those conditions gave the best Faradaic ethene yields. Further optimization attempts of the solvent composition, by adding methanol, which is often done for Kolbe reactions, did not have a positive effect.^[30]^ (Figure S3)

### Influence of 1,4‐Butanediol

During enzymatic hydrolysis of PBS, also 1,4‐butanediol (BD) will be released in addition to succinic acid. To assess the influence of BD, it was subjected to electrochemical tests alone and together with succinic acid. BD alone also yielded ethene, albeit at a low Faradaic ethene yield of 1.0 %. If SA and BD are present simultaneously, the FY decreased from 8.3 % in pure succinic acid to 6.6 %. (see Figure S4) These results suggest that the presence of butanediol in the enzymatic hydrolysis solution will not be a major obstacle for the conversion of succinic acid.

### Influence of Buffer

**Figure 7:**
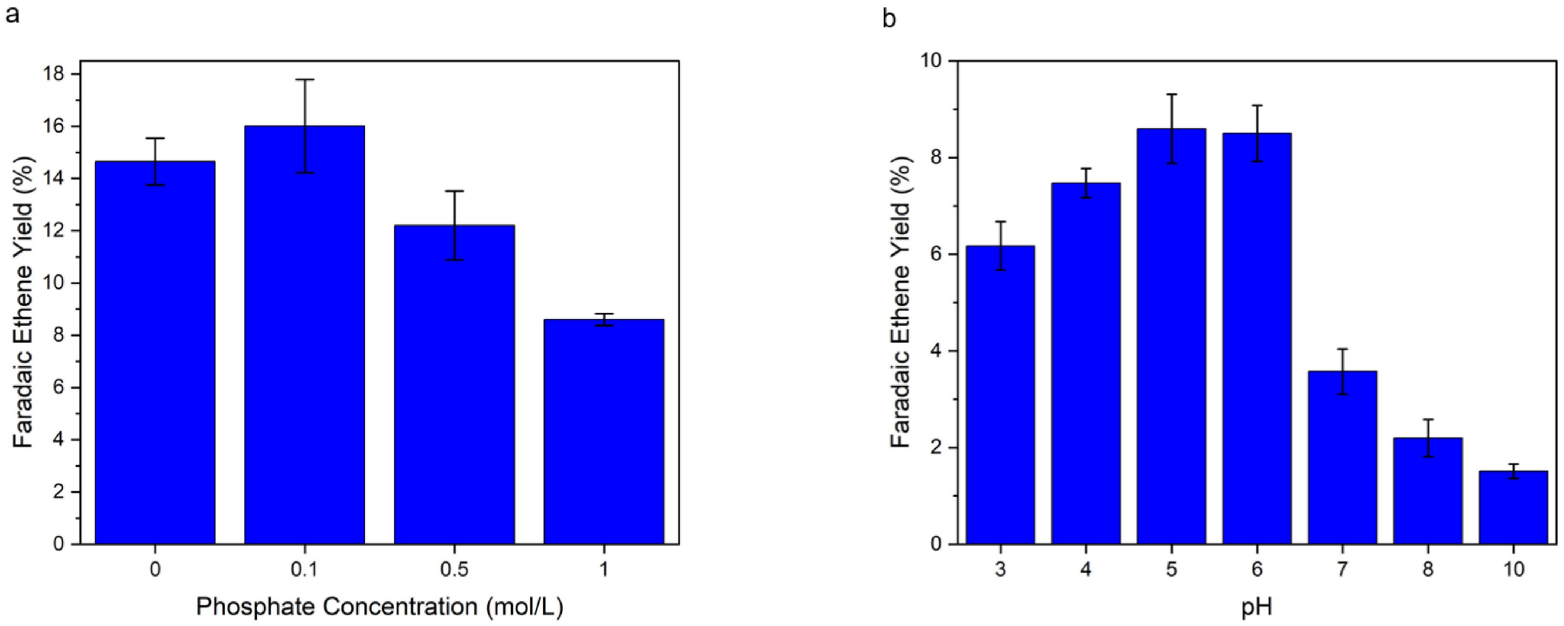
a) Faradaic ethene yields at 25 °C for electrochemical conversion of succinic acid at pH 6; 0.05 mol/L succinic acid, 0, 0.1, 0.5, 1 mol/L phosphate buffer concentration, CA was conducted for 15 min at 1.7 V vs. Ag/AgCl (3 M KCl) after iR compensation; b) Faradaic ethene yields at 25 °C for electrochemical conversion of succinic acid at pH 3,4,5,6,7,8,10; 0.05 mol/L succinic acid, 0.5 mol/L initial phosphate buffer concentration, CA was conducted for 15 min.

As mentioned previously, PBS hydrolysis must be performed in phosphate buffer to avoid a pH drop due to release of succinic acid, which would lead to enzyme deactivation. The direct coupling of enzymatic conversions with electrochemistry is still

rarely investigated. To advance the field of coupled bio‐electrochemical processes, it is crucial to understand the effects of reaction media used in biochemical processes, in electrocatalysis. Therefore, the influence of different phosphate buffer concentrations on the electrochemical decarboxylation was determined. The unbuffered system gave a FY of 14.6 % for ethene. A concentration of 0.1 mol/L potassium phosphate buffer resulted in a similar FY of 16.0 %. For higher phosphate concentrations of 0.5 and 1 mol/L the FY of ethene drops to 12.2 % and 8.6 % respectively, showing that an increased concentration of phosphate diminishes the ethene yield. (Figure 7a) These findings suggest that the enzymatic PBS hydrolysis should be conducted at the lowest phosphate concentration of 0.1 mol/L. The reason for the reduced Faradaic ethene yield at higher phosphate concentrations is presumably a displacement process between succinate and phosphate species at the carbon electrode surface. This hypothesis is further substantiated by the results of molecular dynamics (MD) simulation.

**Figure 8:**
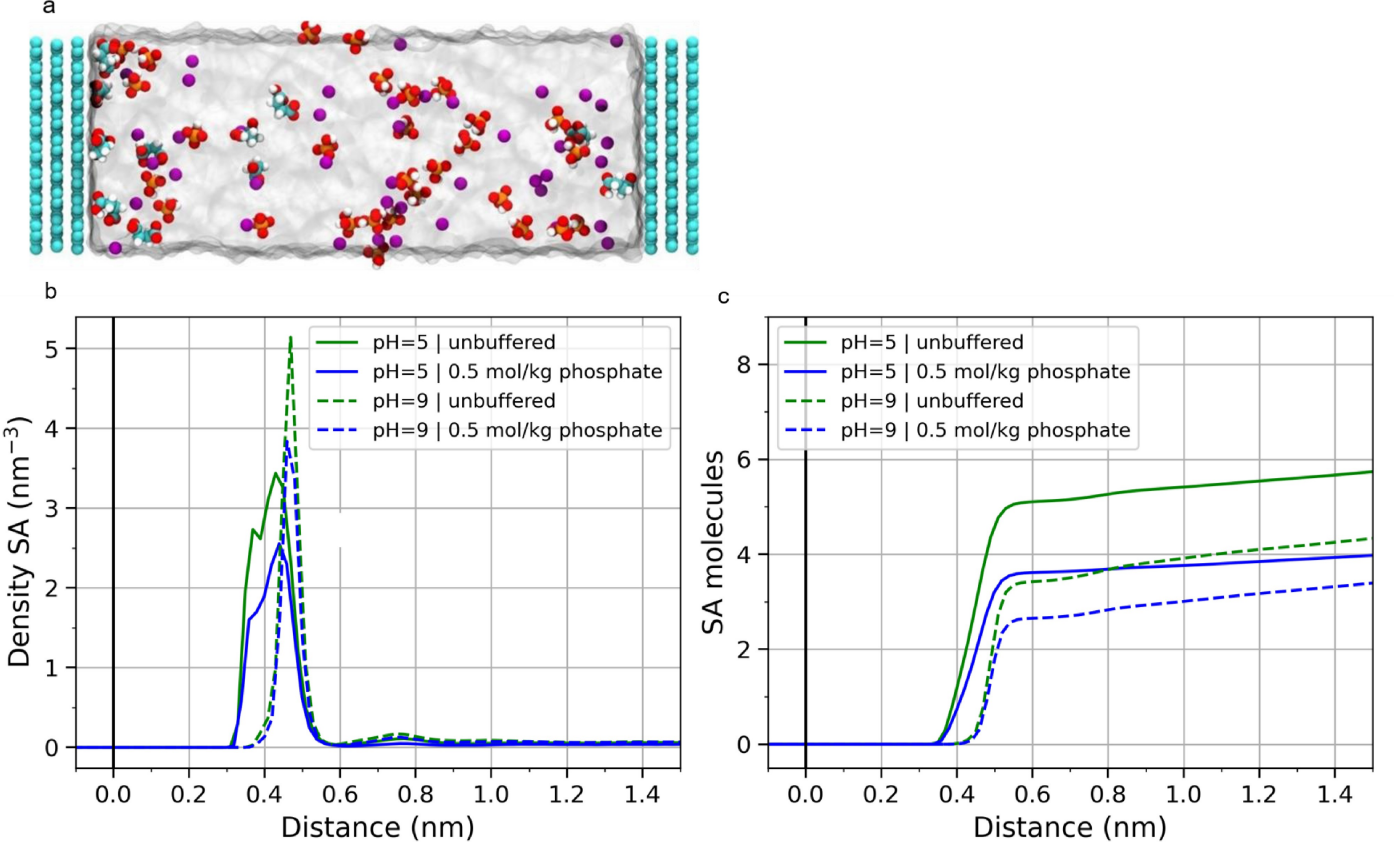
a) Snapshot of MD simulation cell with 0.2 mol/kg SA^−^ and 0.5 mol/kg H_2_PO_4_^−^ (equivalent to pH=5) at 2 V. Carbon is cyan, oxygen red, hydrogen white, phosphorus orange and caesium purple b) The density profile of succinic acid (SA) and c) the cumulative number of SA molecules at different pH and phosphate buffer. The centre of mass of the molecules was used to calculate the distance from the electrode.

LAMMPS^[31]^ (version Nov 2023) with the Electrode Package^[32]^ was used to perform all MD simulations. Four different systems were simulated to represent the system at pH=5 and pH=9 with and without phosphate buffer. The pH values were chosen because SA^−^ (single‐deprotonated succinic acid) and H_2_PO_4_^−^ are the dominant species at pH=5 and SA^2−^ (succinate=double deprotonated succinic acid) and HPO_4_^2−^ are the dominant species at pH=9. Therefore, as an approximation, only these species were required in the MD simulations to describe the pH value accurately. A constant potential of 2 V was applied to all cells. A snapshot of a typical cell is shown in Figure 8a. The addition of phosphate clearly reduces the SA adsorption at both pH levels. (Figure 8b shows the density of SA next to the positively charged electrode. In Figure 8c the cumulative number of SA molecules is plotted)

In the immediate vicinity of the electrode (distance up to 0.6 nm), the number of SA molecules is reduced by 29 % for pH=5 and by 23 % for pH=9 in the presence of phosphate species. An explanation for the reduction is that the negative phosphate ions can screen the applied potential. Therefore, some of the SA ions in the electrochemical double layer (EDL) get replaced by phosphate. This result shows a mechanism, in which the phosphate can reduce the SA concentration at the interface and thus the Faradaic ethene yield, as observed experimentally. This effect could be amplified when SA reacts, as new ions would need to diffuse towards the interface to form the EDL. This could lead to an accumulation of phosphate in the EDL, further diluting the interfacial SA concentration. The MD simulations only take into account a small cell with a perfectly flat graphite electrode. In real electrodes there are pores, functional groups and more, which could lead to further adsorption or pore blocking from the phosphate buffer or side products, reducing the effective surface area and thus the reaction rate.

### pH Optimization

Succinic acid and phosphate species exhibit different states of protonation at different pH values. It can be expected that this will have a significant effect on the formation of the electrochemical double layer and potential adsorption processes during the electrocatalytic step. Therefore, the influence of the pH value was investigated as well. Important for properly assessing the reaction behavior are the pK values of the involved acids which are for succinic acid: pK_1_=4.16 and pK_2_=5.61.^[33]^ For phosphoric acid the values are: pK_1_=2.16, pK_2_=7.21 and pK_3_=12.33.^[34]^ To investigate the influence of different states of the species, pH series in an unbuffered (succinic acid only) as well as in a buffered (succinic acid+phosphate) system were conducted. In the unbuffered system (Figure S5), the maximum Faradaic ethene yield was found to be at pH 4 with 21.2 % FY. The optimum at pH=4 corresponds to the pK_1_ value of succinic acid. At this pH already a relatively large share of all succinic acid species is in the mono‐deprotonated state. Deprotonation can facilitate adsorption on the electrode, promoting ethene formation.^[22]^ Higher pH values are not beneficial because OER becomes more dominant in basic media, decreasing the Faradaic ethene yield for the succinic acid decarboxylation.

For the buffered pH series, test solutions were prepared, that should represent the typical enzymatic PBS hydrolysis solution. The test solutions contain 0.05 mol/L SA and 0.5 mol/L potassium phosphate buffer at pH=8, which is also the pH value used for the enzymatic hydrolysis. For changing the initial pH value of 8, phosphoric acid was added to lower the pH or potassium hydroxide to increase the pH value. Thus, it should be noted that the concentration of phosphate species is not precisely 0.5 mol/L for lower pH values, but in a comparable range. Maximum FY could be achieved at pH 5–6, and therefore deviating from the optimum pH in the unbuffered solution. (Figure 7b) A significant decrease of Faradaic ethene yield (from approx. 8.5 % to 3.6 %) is occurring from pH 6 to pH 7. This shift coincides with the pK_2_ value of phosphoric acid (7.21), meaning that at pH 7, a significantly higher amount of HPO_4_^2−^ will be present in the electrolyte and not merely H_2_PO_4_^−^, as at lower pH values. It can be assumed that the double charged HPO_4_^2−^ will behave differently in the formation of the electrochemical double layer and thereby influence the accessibility and adsorption of succinic acid on the electrode, resulting in an overall decreased Faradaic ethene yield. This assumption was also supported by MD simulations.

When investigating the MD results in Figure 8b and c, further differences between the systems at pH=5 and pH=9 can be spotted. Firstly, the maximum of the succinic acid species at pH 9 is located further away from the electrode than at pH 5. It must be noted that the most prevalent species at pH 9 is the succinate (SA^2−^), while at pH 5 the mono‐deprotonated succinic acid is most prominent (SA^−^). Higher charged ions form a more compact hydration shell. This makes it less favorable for them to adsorb close to the electrode, as they need to disperse part of their hydration shell. This effect is also demonstrated for HPO_4_^2−^ and H_2_PO_4_^−^ (Figure S6). Second, there are fewer SA^2−^ molecules than SA^−^ adsorbed on the electrode. In the immediate vicinity of the electrode (distance up to 0.6 nm) the SA‐species number is 33 % lower for the unbuffered system and 27 % lower for the buffered system. This can also be explained with the screening in the EDL. As SA^2−^ is doubly charged, fewer molecules are needed to screen the potential. This is also consistent with the experiments, where the Faradaic ethene yield is lower at high pH values.

### Long‐Term Reaction of Enzymatic Hydrolysis Solution

After determining the optimal electrochemical conditions (0.1 mol/L potassium phosphate, pH 6, 25 °C and 2.5 V vs. Ag/AgCl (3 M KCl)), the reaction solutions obtained from enzymatic PBS hydrolysis were tested. There was not a significant difference in composition between hydrolysis solution of pristine PBS or waste cups. The increased voltage of 2.5 V, compared to lower voltages during optimization experiments, was chosen to overcome the higher voltage drop due to the higher solution resistance resulting from the lower K‐PO_4_ concentration, compared to the optimization experiments. No ethene peak in GC analysis was observed during a blank measurement (buffer without succinic acid). The enzymatic hydrolysis solutions were filtrated, and their pH value was set to 6 by adding phosphoric acid. Otherwise, no changes were made before the electrochemical test, allowing utilization of the enzymatic hydrolysate with minimum preparation or pretreatment. The solutions were tested under constant potential electrolysis conditions (2.5 V vs Ag/AgCl) for 8 h in a three‐electrode batch setup (see experimental description for details).

**Figure 9:**
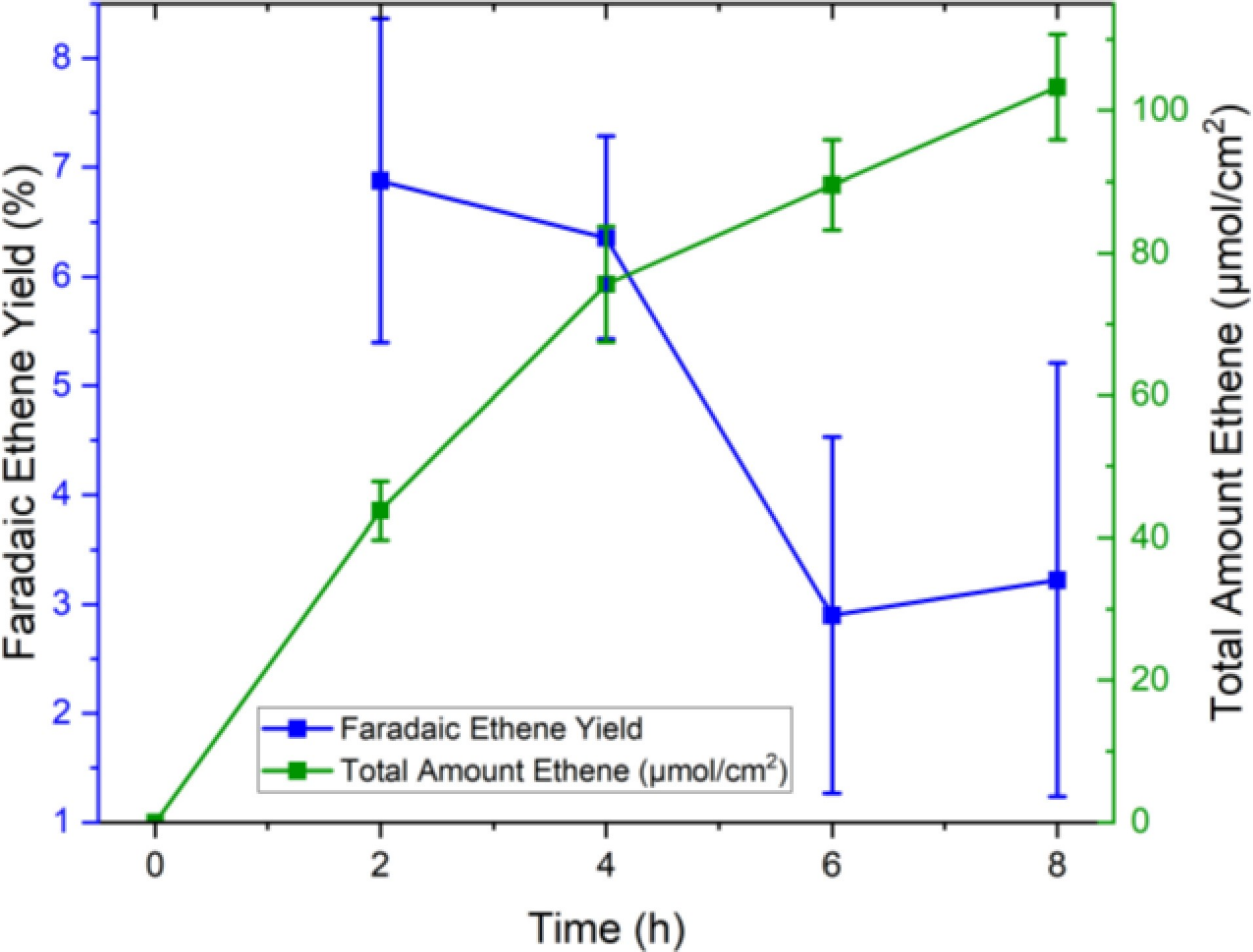
Faradaic ethene yields and total amount of ethene produced at 25 °C; enzymatic hydrolysis solution contained 0.028 mol/L succinic acid, 0.1 mol/L K‐PO_4_buffer at pH=6, 4 CVs were performed at a scan rate of 80 mV/s from 0–2.5 V vs Ag/AgCl before conducting CA for 8 hours at 2.5 V vs Ag/AgCl (3 M KCl).

The headspace of the batch reactor was sampled with a gas tight syringe in continuous time intervals (2 h), using GC for detecting the main reaction products, ethene and H_2_. Constant evolution of ethene over the whole reaction time was seen indicating stable catalytic behavior (Figure 9). For these long‐term experiments, several factors have to be considered that can cause additional statistical variations for the Faradaic ethene yield. For example: Vigorous stirring of the reaction solution is believed to have caused fluctuations of the distance between working and reference electrode. This influences the actual voltage applied to the working electrode. Furthermore, soaking of the carbon felt can alter the structural integrity of the electrodes. The Faradaic ethene yields calculated at individual time points, are nevertheless decreasing over time, which can be explained by diminishing substrate concentration and potential deactivation of the carbon felt electrode. After 8 h a total amount of 91 μmol/cm^2^ ethene was formed with an average Faradaic ethene yield (over the whole reaction time) of 4.9±1.1 %. As mentioned previously, hydrogen was formed as additional value‐added cathodic product, with a Faradaic hydrogen yield of 51.2±8.9 %. HPLC measurements revealed that on average 76 % of SA was in general converted including other reaction pathways. The blank test revealed the formation of CO_2_ from carbon felt oxidation even in absence of SA. In the long‐term experiments 86.4 % of all CO_2_ formed can be attributed to the decarboxylation reaction. The remaining amount is most likely a result of carbon felt oxidation and substrate overoxidation (Table S3). In total 21.7 % of SA in PBS  were converted into ethene, the majority of the remaining SA is converted in carboxylic acid reaction intermediates (acrylic acid, propanoic acid or formic acid). 77 % of BD remained after the electrochemical reaction in the liquid phase. This indicates preferred electrochemical conversion of SA. An explanation for the high selectivity towards SA is that neutral BD is present in lower concentrations in the anodic double layer, compared to the charged SA species. Degradation of the carbon felt electrode was determined by Raman and XPS analysis of the felt after the reaction. For Raman measurements the I_D_/I_G_ ratio increased from 0.95 for the pristine felt to 1.57 for the spent felt, suggesting the formation of defects in the spent felt (Figure S7–S9). Furthermore, XPS analysis of the spent felt was conducted and compared to pristine felt (Figure S10 and Figure S11). While the sp^2^ peak of the C1s region made up 77 % of peak area in the pristine felt, it only accounted for 35 % of total peak area of the spent felt. Instead, peaks corresponding to oxidized carbon species (i. e. COH, CHO and COOH) are more dominant in the XPS spectra of the spent felts and this result further confirms that the felt itself is also being oxidized.

## Conclusions

In this work, a two‐step process was developed for chemical recycling of PBS into ethene and 1,4‐butanediol, while generating H_2_ as additional cathodic product. The first step was the enzymatic hydrolysis of PBS, where using HiC for enzymatic hydrolysis proved to be more efficient compared to TIL. Systems in K‐PO_4_ buffer allowed close to full conversion of the PBS starting material (10 mg PBS dissolved in 2 mL enzyme solution and 4 to 10 U of enzymatic activity per mg PBS), even at low buffer concentrations of 0.1 mol/L. Furthermore, the selective hydrolysis of the PBS coating in real waste cups was demonstrated. In electrochemical tests with model systems the detrimental effect of the K‐PO_4_ buffer as well as the influence of pH values were determined. MD simulations supported the mechanistic hypothesis, that different phosphate species compete with the succinate intermediates for adsorption on the positively charged electrode. These findings highlight the importance of understanding the fundamental processes in electrolyte solutions and will support the development of improved bio‐electrochemical cascade processes in the future. The electrochemical conversion of the PBS derived succinic acid could be also demonstrated for reaction times of up to 8 hours, using enzymatic hydrolysis solutions, that could be directly utilized, after adjusting the pH value. The developed process demonstrated for the first time the chemical recycling of PBS into the value‐added product ethene (with H_2_ as cathodic secondary product), while the majority of 1,4‐butanediol remains in the aqueous solution. The process is performed in aqueous solutions and benign temperatures, avoiding the necessity of costly product separation, as ethene and H_2_ are released as gas, and 1,4‐butanediol remains in the reaction solution.

## Experimental

### Chemicals, Substrates, Enzymes

Dipotassium hydrogen phosphate (K_2_HPO_4_), potassium dihydrogen phosphate (KH_2_PO_4_) and para‐nitrophenyl‐butyrate (pNPB) for enzymatic hydrolysis were purchased from Sigma‐Aldrich or Carl Roth. KOH (≥85 %) and 1,4‐butanediol were obtained from Carl Roth (99 % for synthesis). SIGRACELL GFD 4.6 carbon felts (CF) were provided from SGL Carbon.

All other chemicals and reagents used in this work were of analytical grade and used without further purification if not otherwise specified. *Humicola insolens* cutinase (HiC) was purchased from STREM chemicals (BISCHHEIM, France, product code 06‐3135, Novozym 51032, CAS Number: 9001–62‐1). *Thermomyces lanuginosus* lipase (TlL) was purchased from Sigma‐Aldrich (Vienna, Austria).

Polybutylene succinate was purchased from Resonac (formerly Showa Denko, Bionolle™ 3001 MD), and PTT MCC Biochem Co., type FZ71 (biodegradable Polymer “BioPBS™) Some of the materials were processed via ultra‐centrifugal‐mill to a particle size <1 mm. The BioPBS butterfly cup, which consists of cellulose and PBS was purchased from aboutwater GmbH.

### Enzyme Characterization: Protein Concentration and Activity Assay

For enzyme concentration determinations via Bradford assay, samples (10 μL of diluted enzyme solution, bovine serum albumin protein (BSA) standards and blanks) were placed in triplicates in a transparent 96‐well Greiner plate. After adding 200 μL of diluted Bradford reagent (Bio‐Rad laboratories GmbH, Vienna, Austria), a 5 min incubation at 20 °C was carried out while shaking. Absorbance was measured at 595 nm via Tecan plate reader. Due to the reference of BSA protein standards, the protein concentration of present enzymes could be calculated subsequently.

For the determination of cutinase activity, para‐nitrophenyl‐butyrate (p‐NPB) was used. 20 μL of the enzyme (prediluted in defined buffer solutions at the enzymes optimal pH and a buffer capacity of 100 mM, to 1 : 100 and 1 : 1000) were placed in triplicates in a transparent 96‐well plate. Afterwards, 200 μL of the substrate (diluted in 2‐methyl‐2‐butanol and buffer of incubation) were added to both the blanks (water/buffer) and the enzyme dilutions. Absorbance measurements were performed at 405 nm for 10 min at 30 °C every 18 seconds. Corresponding to the fraction of enzyme necessary to hydrolyse 1 μmol of substrate per minute, the activity was expressed in Units.

### Enzymatic Hydrolysis of PBS

A total of 10 mg of ultra‐centrifugal milled PBS was incubated in triplicates in 2 mL of 1 M and 0.1 M potassium‐phosphate buffer, tap water (TW) or Milli‐Q (MQ) water at pH 8.0, containing HiC or TlL at a concentration of 40 U mL^−1^. As real waste substrate, 2 g shredded material of a commercial PBS cellulose cup was incubated in 40 mL of similar buffer composition. For preparation of 1 M potassium‐phosphate buffer at pH 8, two 2 M stock solutions (54.44 g KH_2_PO_4_/ 69.67 g K_2_HPO_4_, each per 200 mL MQ) were mixed in a ratio of 2.65 mL KH_2_PO_4_ stock and 47.35 mL K_2_HPO_4_ stock together with 50 mL MQ water. A 10 times diluted version was prepared to gain a concentration level of 0.1 M. The solutions were incubated at 65 °C and 150 rpm. Reactions were stopped at various time points (2, 4, 24, 72, and 168 h) removing the enzyme (or blank) solution and freezing the vial with the already obtained monomer concentrations before carrying out HPLC sample preparations and various characterizations, e. g. pH measurements.

### Quantification of Hydrolysis Products

After each timepoint, the recovered released monomers were quantified via high‐performance liquid chromatography (HPLC) (Agilent Technologies, 1260 Infinity) equipped with crosslinked polystyrene column (ION‐300, Concise separationsTM). For the quantification of 1,4‐butanediol and succinic acid, a refractive index detector was used. For removal of proteins prior to HPLC analysis, Carrez clarification based on potassium hexacyanoferrate(II) trihydrate and zinc sulfate heptahydrate reagents, was performed. These two reagents were added step‐by‐step to the hydrolysates and allowed to rest, respectively, for 1 and 10 min. Before filtering the clear supernatant into an HPLC vial, centrifugation steps were performed (30 min, 1400 rpm, 4 °C). The system was used with a refractive index detector (Concise separationsTM IC SEP‐ION‐300) and run with 0.01 N H_2_SO_4_, at a flow rate of 0.325 mL min^−1^ and 45 °C. Hydrolysis product concentrations were calculated based on standard calibrations with both monomers (1,4‐butanediol/succinic acid).

### Fourier Transform Infrared (FT‐IR) Spectroscopy

Fourier transform infrared (FT‐IR) spectroscopy measurements were carried out for assessing the hydrolysis of the ester bonds by enzyme activity. Once retrieved from the incubator, each PBS film was first washed with 2 mL Triton solution 5 g/L, then with 2 mL Na_2_CO_3_ 10 mM and finally rinsed twice with Milli‐Q water. For each of the washing steps, samples were maintained at room temperature and shaken at 400 rpm for 10 minutes, then they were placed in a 48‐well plate and left to dry. FT‐IR sampling was performed using a FT‐IR spectrometer (Spectrum 100, PerkinElmer). All samples were scanned in the spectrum range from 4000 cm^−1^ to 650 cm^−1^ with a scan accumulation of 40. Results were then converted into absorbance values, corrected with the background and normalized for the whole spectrum.

### Scanning Electron Microscopy (SEM)

The trend of PBS film morphology was qualitatively assessed with Scanning Electron Microscopy (SEM). Control PBS (without any enzymatic treatment) and enzymatically hydrolysed PBS films (after 2 and 4 h) were characterized. Polymers were cut in 0.5 cm×0.5 cm squares for performing the enzymatic incubation. Afterwards, the films were washed in three subsequent steps for removal of surface impurities (Triton X‐100, Na_2_CO_3_ and final rinsing with ultrapure water). In addition, the fracture surface was sputter coated with a 2 nm layer of platinum to provide sufficient electrical conductivity. All SEM images were acquired collecting secondary electrons on a Hitachi 3030 TM (Metrohm INULA GmbH, Austria) using 5 keV.

### Electrochemical Measurements & Gas Quantification

Electrochemical measurements (i. e. cyclovoltammetry (CV), potentiostatic electrochemical impedance spectroscopy (PEIS), linear sweep voltammetry (LSV) and chronoamperometry (CA)) were conducted with a BioLogic 150e potentiostat. If not mentioned otherwise, the measurements were performed at room temperature (25 °C).

The electrocatalytic measurements were conducted in a 50 mL three‐necked flask filled with 30 mL of the respective solution. The test solutions contained 0.05 mol/L succinic acid. The electrocatalytic tests were performed in a three‐electrode configuration with carbon felt (active electrode area 2 cm^2^) serving as working electrode, an Ag/AgCl reference electrode (3 mol/L KCl, ItalSens) and a Pt foil counter electrode. The flask was sealed with rubber septa to ensure gas tightness of the setup. The solution was purged for 15 minutes with N_2_, before commencing electrochemical tests. All potentials are given vs. Ag/AgCl (3 M KCl). No iR compensation was performed for the experiments, except for the screening of the phosphate buffer concentration and the pH screening in unbuffered solutions (due to large variations in electrolyte resistance, caused by different buffer concentrations).^[35]^

To ensure reproducible wetting of the electrode 4 CVs in the range from 0–2.5 V vs Ag/AgCl (3 M KCl) with a scan rate of 80 mV/s were performed initially. PEIS was conducted (at 1.0 V from 100 mHz ‐1 MHz) to determine the solution resistance and to perform full iR compensation for the buffer and unbuffered pH optimization tests. Then CA was conducted for 15 minutes.

For all experiments, the gaseous products were collected in the headspace of the 3‐necked flask, serving as reaction vessel. For 8 h extended experiments, additionally a 500 mL glass volume‐extension vessel was used to increase the available gas volume. After 4 hours of CA, the setup was flushed with N_2_ then again 4 CVs and additional 4 hours of CA were conducted.

After CA, a sample from the headspace of the three‐necked flask was taken with a gas tight syringe and sampled into the gas chromatograph (GC) for analysis of the gas sample. The comparably large volume of the reaction vessel and height difference between the electrode and the top of the extension vessel, can lead to limited gas diffusion and potentially delay GC responses. For gas analysis, a Shimadzu Nexis 2030 gas chromatograph with He as carrier gas equipped with a ShinCarbon ST micropacked GC column for separation and a barrier‐ion discharge detector (BID) for detection was used. The pressure in the inlet system was 226.8 kPa for the first 2.5 min, then ramped up to 390.1 kPa at a rate of 15.2 kPa/min and held for 5.95 min. Next the pressure was increased to 405.1 kPa at a rate of 11.2 kPa/min and held for 5.42 min. The temperature of the inlet system was 150 °C. The temperature program of the column was as follows: holding 50 °C for 2.5 min, then ramping up to 250 °C at a rate of 10 °C/min, then ramping up to 270 °C at 9 °C/min and held for 3 min at 270 °C.

From the ethene concentration determined by the total amount of ethene in the flask the Faradaic yield (FY) was calculated, following Equation 1:

(1) $$\mathrm{FY}=\frac{n_{Ethene}\cdot 2\cdot 96485}{Q}\cdot 100$$

where $n_{Ethene}$ is the total amount ethene produced [mol], 2 is the number of electrons transferred per succinic acid molecule to produce ethene [−], 96485 is the Faraday constant [C/mol] and Q is the total amount of transferred charge obtained from CV and CA experiments [C].

### High Performance Liquid Chromatography (HPLC)

The liquid phase was analyzed post‐electrolysis using a Shimadzu LCMS‐2020 liquid chromatograph. The analysis was conducted with a Shim‐pack SCR‐102H ion exchange column (300×8.0 mm, 7 μm) maintained at 70 °C. Detection was either performed with a UV SPD‐40 V detector set at a wavelength of 220 nm or a refractive index detector. The mobile phase (0.1 % H_3_PO_4_ in H_2_O) flow rate was maintained at 0.8 mL/min.

### MD Settings

Starting positions were randomly generated with Packmol^[36]^ and the data file was created with Moltemplate.^[37]^ The solvent was 3333 water molecules and for the anions 12 SA molecules and, when added, 30 phosphate molecules were used. To achieve better sampling, the SA concentration was about four times higher than in the experiment. This should not be significant as the interactions between SA molecules is small. Cations were added so the cell was charge neutral. In general, it is not possible to simulate explicitly hydrogen ions present in the experiment with classical molecular dynamics. We choose large caesium ions, as with smaller cations the formation of clusters was observed for the system with 0.5 mol/kg HPO_4_^2−^. As electrodes 3 layers of graphene with an area of 3.4×3.4 nm and a distance of around 10 nm were used.

A constant potential was applied between the Electrodes with the Electrode package.^[32]^ The Electrode package describes the graphite as perfectly polarisable. A PPPM k‐space solver with a relative accuracy of 10^−6^ computed the long‐range interactions. The system was periodic in the electrode plane and a PPPM correction for the slab geometries was used.^[38]^ A thermostat controlled the temperature by global velocity rescaling with Hamiltonian dynamics at 298 K.^[39]^ Each system was simulated 10 times with independent starting positions for 25 ns, where the last 15 ns were used for analysis.

To calculate the interactions the following force fields were used for SPC/E water,^[40]^ graphite^[41]^, SA and phosphate^[42]^ and caesium.^[43]^ The partial charges for SA were calculated with resp fits^[44]^ and the partial charges for phosphate provided by^[45]^ Lorentz‐Berthelot mixing rules were used for the force fields and the non‐bonded interactions had a cutoff of 1 nm. All hydrogen bonds and the angle in water was held rigid with the shake algorithm and a time step of 1 fs was used.

## Conflict of Interests

The authors declare no conflict of interest.

1

## Supporting information

As a service to our authors and readers, this journal provides supporting information supplied by the authors. Such materials are peer reviewed and may be re‐organized for online delivery, but are not copy‐edited or typeset. Technical support issues arising from supporting information (other than missing files) should be addressed to the authors.

Supporting Information

## Data Availability

The data that support the findings of this study are available from the corresponding author upon reasonable request.
