# Supplementary material for: Demonstration of a Chemical Recycling Concept for Polybutylene Succinate Containing Waste Substrates via Coupled Enzymatic/Electrochemical Processes
Source: ChemSusChem. 2025 Feb 19;18(10):e202402515. doi: 10.1002/cssc.202402515 (PMC12094149; doi:10.1002/cssc.202402515)
Supplement: Supplementary file 1 — Supporting Information [file CSSC-18-e202402515-s001.pdf]

ChemSusChem

Supporting Information

Demonstration of a Chemical Recycling Concept for Polybutylene Succinate Containing Waste Substrates via Coupled Enzymatic/Electrochemical Processes

Richard Buchinger, Sabrina Bischof, Ole Nickel, Vanessa Grassi, Jasmin Antony, Markus Ostermann, Soniya Gahlawat, Markus Valtiner, Robert Meißner, Georg Gübitz, and Christian M. Pichler*

Demonstration of a Chemical Recycling Concept for Polybutylene Succinate containing Waste Substrates via Coupled Enzymatic/Electrochemical Processes

Richard Buchinger,[a] Sabrina Bischof,[b] Ole Nickel,[d],[e] Vanessa Grassi,[b] Jasmin Antony,[b] Markus Ostermann,[c] Soniya Gahlawat,[c] Markus Valtiner,[a],[c] Robert Meißner,[d],[e] Georg Gübitz[b] and Christian M. Pichler*[a],[c].

[a] R. Buchinger, Prof. M. Valtiner, Dr. C. M. Pichler
Institute of Applied Physics, Vienna University of Technology
1040 Vienna, Austria
E-mail: pichlerc@iap.tuwien.ac.at

[b] S. Bischof, V. Grassi, J. Antony, Prof. G. Gübitz
Institute of Environmental Biotechnology, University of Natural Resources and Life Sciences Vienna
3430 Tulln, Austria

[c] Dr. M. Ostermann, Dr. S. Gahlawat, Prof. M. Valtiner, Dr. C. M. Pichler
Center for Electrochemical Surface Technology GmbH
2700 Wr. Neustadt, Austria

[d] O. Nickel, Prof. R. Meißner
Institute for Modeling of Soft Matter, Hamburg University of Technology
21073 Hamburg, Germany

[e] O. Nickel, Prof. R. Meißner
Institute of Surface Science, Helmholtz-Zentrum Hereon
21502 Geesthacht, Germany

PBS Hydrolysis

IR spectroscopy

The peaks indicating PBS hydrolysis were: the peak at 917 cm^{-1}, corresponding to the –C–OH bending in the carboxylic acid groups of PBS (a); the peaks between 1044-1046 cm^{-1}, corresponding to –O–C–C– stretching vibrations in PBS (b); the peaks in the range of 1144–1264 cm^{-1} and at the 1710–1713 cm^{-1} region, corresponding respectively to the stretching of the –C–O–C– group in the ester linkages of PBS (c) and to the C=O stretching vibrations of ester groups in PBS (d), and the peaks assigned to the vibrations of $–CH_2$ groups in the PBS main chains (e) at 1330 and 2945 cm^{-1}. [36] Results show that peaks corresponding to the groups –C–OH (a), –O–C–C– (b), –C–O–C– (c) and $-CH_2$ (e) decrease during the incubation with the enzymes, confirming that ester bonds undergo hydrolysis. For these measurements, 0.09 mg/mg PBS of 1,4-butanediol and 0.07 mg/mg PBS of succinic acid were released after 4 h.

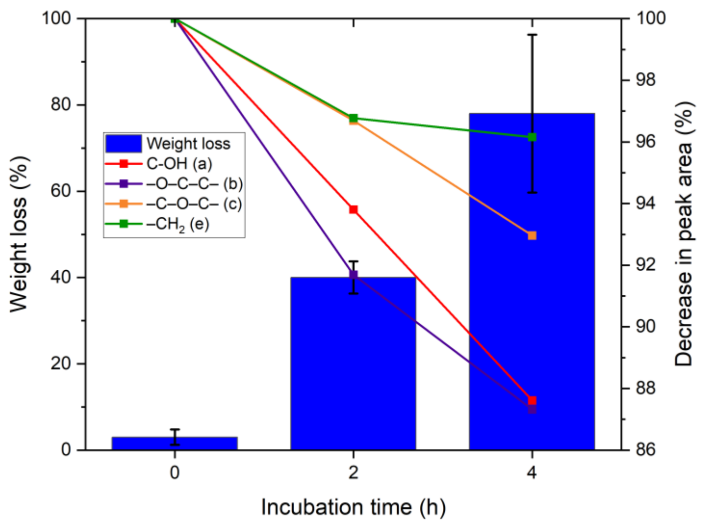

Fig. S1: Enzymatic degradation of PBS films- FT-IR analysis to monitor the decrease of significant peaks indicating plastic degradation + weight loss (%) of PBS films after drying

Hydrolysis in different media

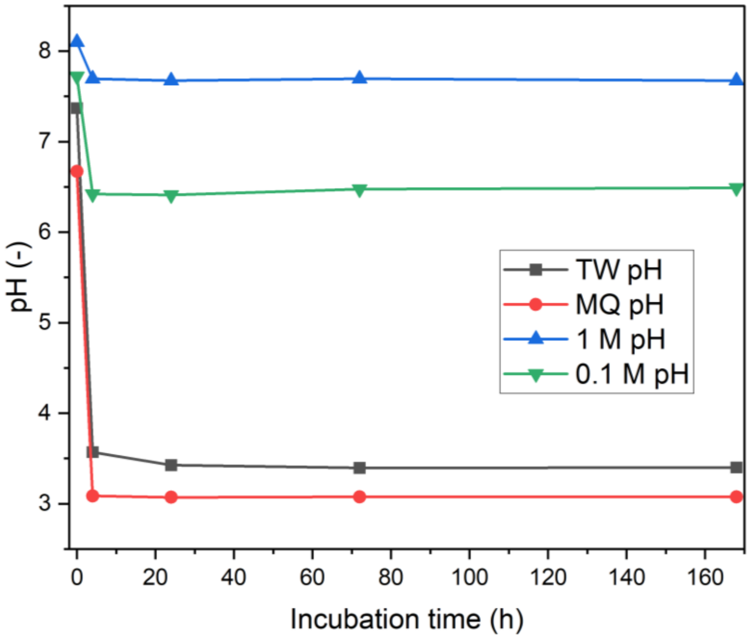

Fig. S2: Enzymatic hydrolysis of PBS by HiC – pH changes upon hydrolysis mediated by 4 U HiC/mg PBS in different media

Hydrolysis of paper cup

Table S1: Product concentrations of BD and SA after incubation of PBS covered paper cups in HiC solution, 2 g "cup" in 40 mL enzymatic hydrolysis solution (0.1 M potassium-phosphate buffer and 40 U mL^{-1} enzyme concentration)

Time (h)	BD (mg/mL)	SA (mg/mL)
0	0.00±0.00	0.00±0.00
24	2.32±0.03	3.42±0.02
72	2.35±0.08	3.50±0.06
168	2.55±0.06	3.65±0.04

Electrochemical Optimization

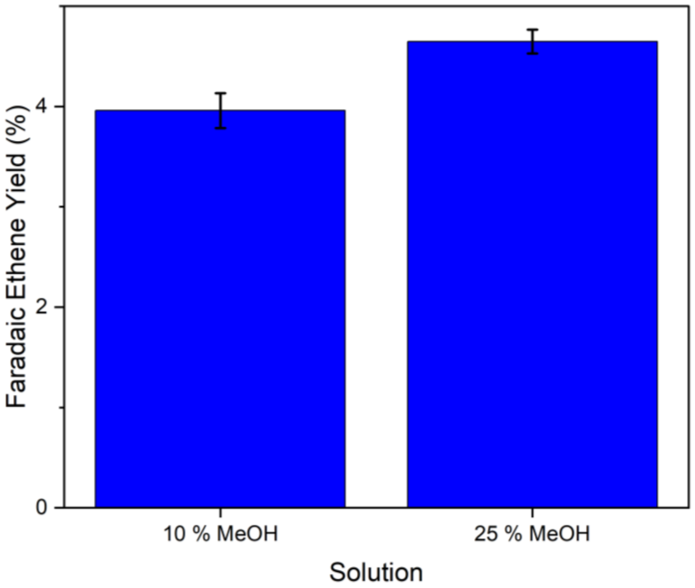

Fig. S3: Faradaic yields towards ethene of electrochemical conversion of succinic acid to ethene at 25 °C; 50 mmol/L succinic acid, 0.5 mol/L phosphate buffer, 10 % and 25 % methanol (MeOH) at pH=6, initial CVs for electrode wetting were performed at a scan rate of 80 mV/s from 0-2.0 V vs Ag/AgCl, then CA was conducted for 15 min at 2.0 V

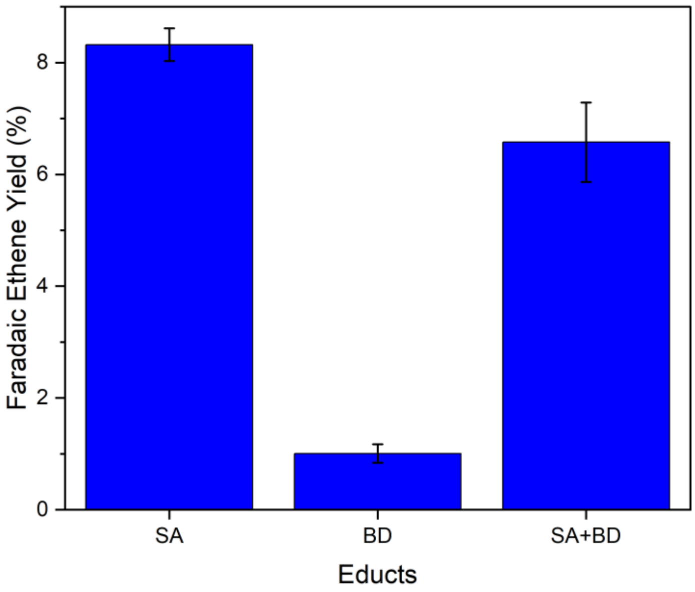

Fig. S4: Faradaic yields towards ethene of electrochemical conversion of 1,4-butanediol and succinic acid alone and for both educts present to ethene at 25 °C; 50 mmol/L succinic acid, 0.5 mol/L phosphate buffer at pH=6, initial CVs for electrode wetting were performed at a scan rate of 80 mV/s from 0-2.0 V vs Ag/AgCl, then CA was conducted for 15 min at 2.0 V

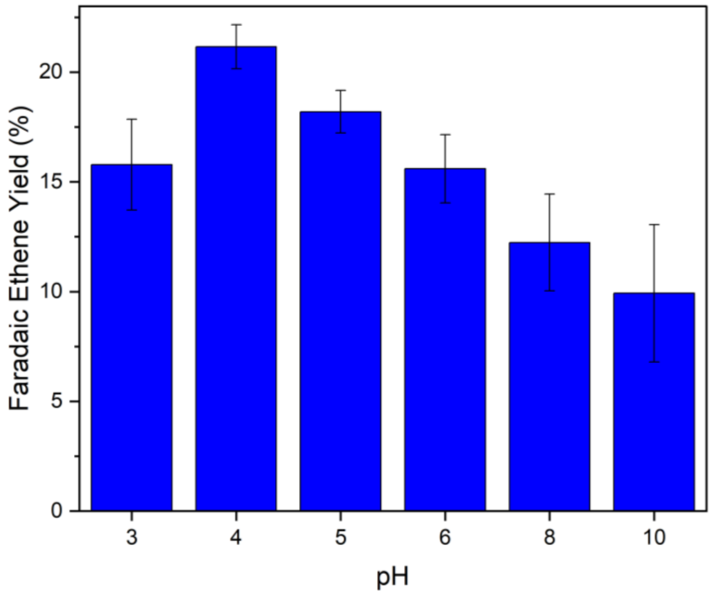

Fig. S5: Faradaic yields towards ethene of electrochemical conversion of succinic acid to ethene at 25°C; 50 mmol/L succinic acid at pH=3,4,5,6,8,10; pH was set by adding KOH (otherwise no other buffer was used); initial CVs were performed at a scan rate of 80 mV/s from 0-2.3 V vs Ag/AgCl, then CA was conducted for 15 min at 1.7 V after iR compensation

MD Phosphate Adsorption

In Fig. S6 the phosphate density is plotted for the systems in the main text. $H_2PO_4^-$ mostly adsorbs at a distance around 0.4 nm, corresponding to the first water layer, while HPO_4^{2-} adsorbs at a distance around 0.6 to 0.7 nm in the second water layer. The difference between the species is probably because the doubly charged molecule has a strongly bound solvation shell, which it would have to disperse to adsorb directly next to the electrode. In comparison to SA (Figure 8a) few phosphate molecules are adsorbed, further showing that the phosphate is not crowding the surface and is only replacing some of the negative SA ions in the EDL.

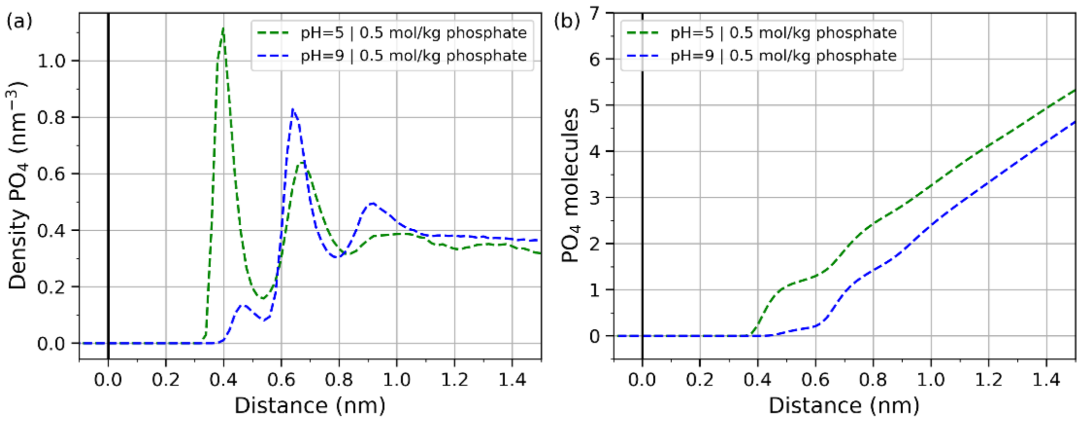

Fig. S6: Density profile of phosphate species ($H_2PO_4^-$ at pH 5 and HPO_4^{2-} at pH 9)

Long-term experiment

Table S2: Amounts of SA and BD before and after electrochemical long-term experiments

Amount SA before reaction (µmol)	Amount SA after reaction (µmol)	Amount BD before reaction (µmol)	Amount BD after reaction (µmol)
838±26	206±171	705±18	544±124

Table S3: Overview Faradaic Yields, total amounts of gases produced and transferred charge

Experiment	FY H_2 (%)	FY C_2H_4 (%)	Total amount H_2 (µmol)	Total amount CO_2 (µmol)	Total amount C_2H_4 (µmol)	Q (C)
1	62.5	5.3	2306	414	196	712
2	40.7	6.0	752	252	112	356
3	50.4	3.3	3602	600	239	1380

A significant part of succinic acid is converted into liquid phase products such as acrylic acid, propanoic acid, acetic acid, adipic acid and formic acid. Exact quantification of those products was not attempted due to insufficient peak separation.

Raman spectroscopy of spent felt

Experimental

Raman spectroscopy was used to determine the defect density in the graphitic fibers. The spectra were recorded using a Horiba LabRam Aramis, equipped with a 532 nm laser, a 600 gr mm^{-1} grating and a 50x magnification objective. Measurements were recorded in 4 spots. Fitting was executed with the software OriginPro according to the fitting protocol of Claramunt et al. using 2 Gaussian (D*, D'') and 3 Voigt-peaks (D, G, D'). [1] An example of a fitted spectrum is shown in Fig. S7.

Results

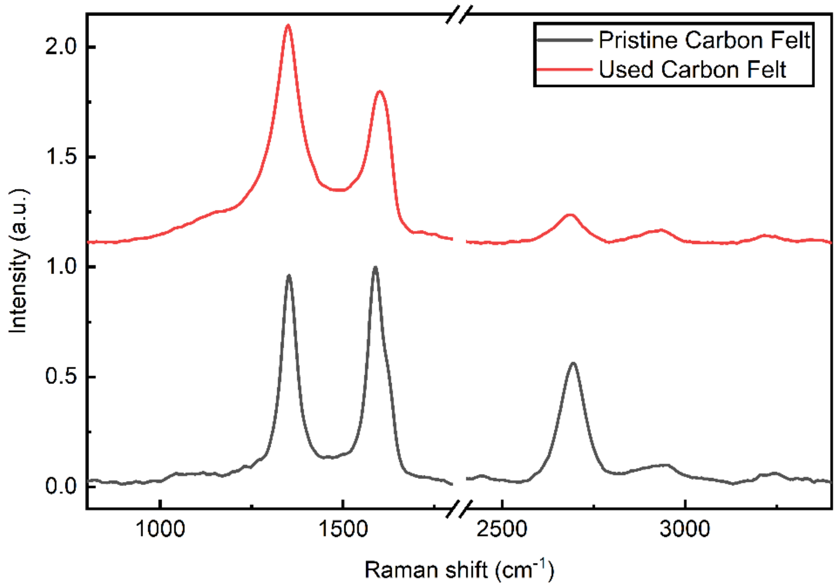

Fig. S7: Raman spectra of pristine and spent felt

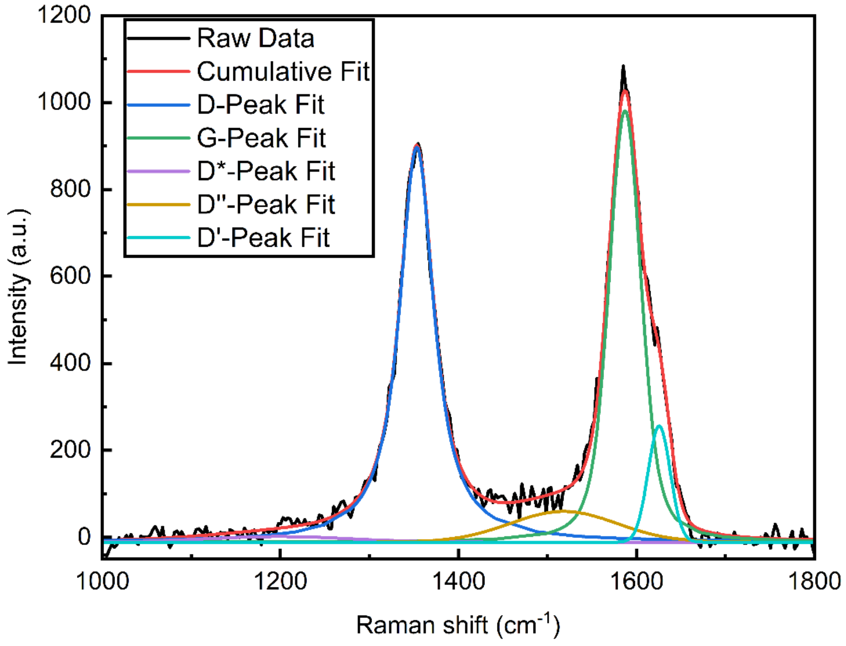

Fig. S8: example of a fit of the Raman spectra

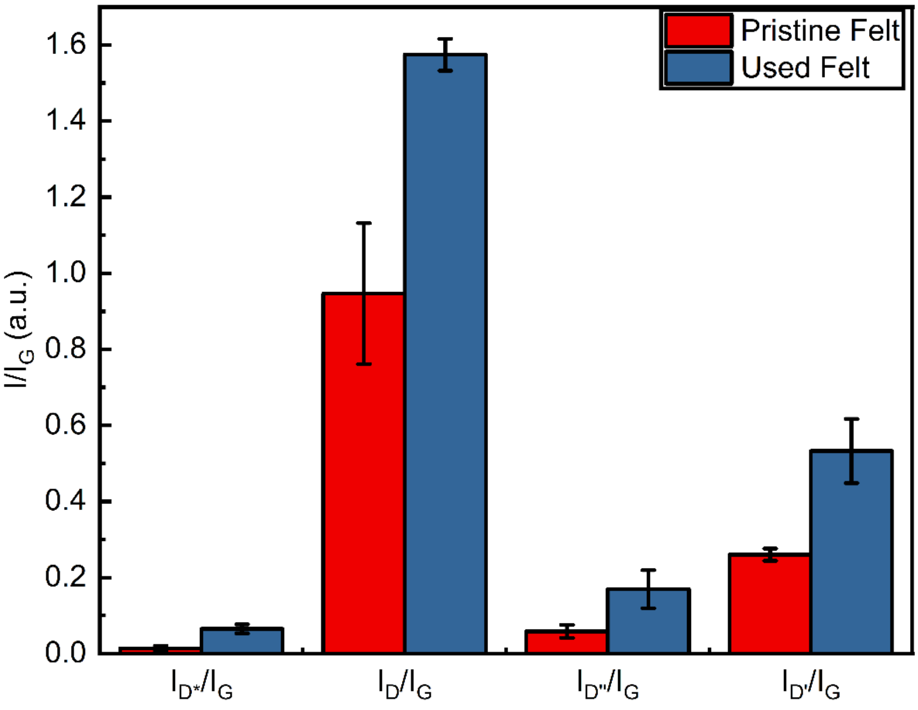

Fig. S9: Ratios of different bands for pristine and spent felt

XPS of spent felt

Experimental

X-ray photoelectron spectroscopy (XPS) measurements were performed using a Versa Probe III spectrometer (Physical electronics GMBH) at the ELSA cluster TU Vienna. Monochromated Al Kα (1486.6 eV) was used as the radiation source, with the beam diameter set to 100 µm and the beam voltage to 15 kV. The samples were mounted on a polymer tape and measurements were performed using an E−I neutralization gun to compensate for the charging effect. Survey scans of all samples were recorded at a pass energy of 140 eV and a step size of 0.125 eV. High-resolution core level spectra were recorded at a pass energy of 27 eV and a step size of 0.05 eV.

CasaXPS was used to process the spectra. The C 1s spectra were corrected with a U 3 Tougaard-type background. sp^2 hybridized carbon was fitted with an asymmetric peak shape, while the remaining species were deconvoluted with Gaussian-Lorentzian peak shapes. Their position was determined with respect to sp^2 carbon, with their FWHM restricted to sp^3 carbon. All spectra were binding energy corrected to the sp^2 carbon signal at 284.4 eV.

Results

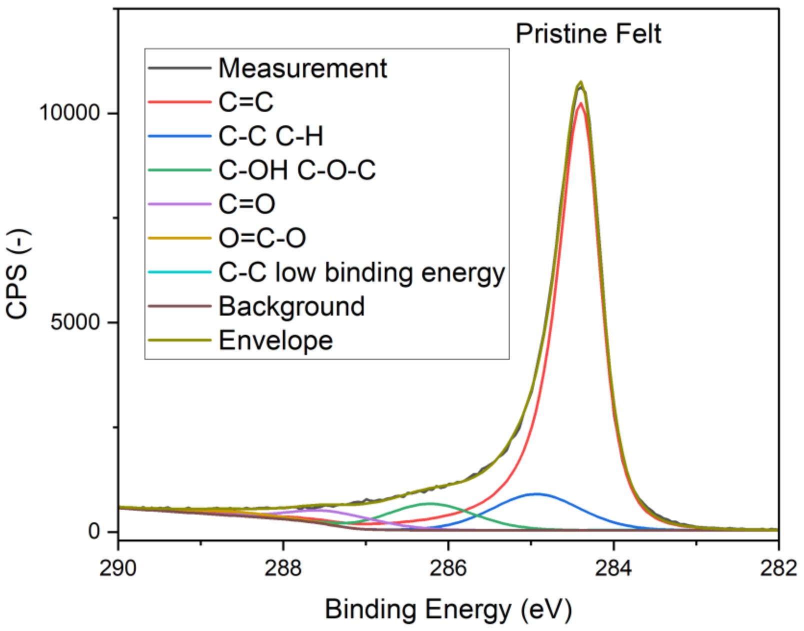

Fig. S10: XPS spectrum and fit of pristine carbon felt

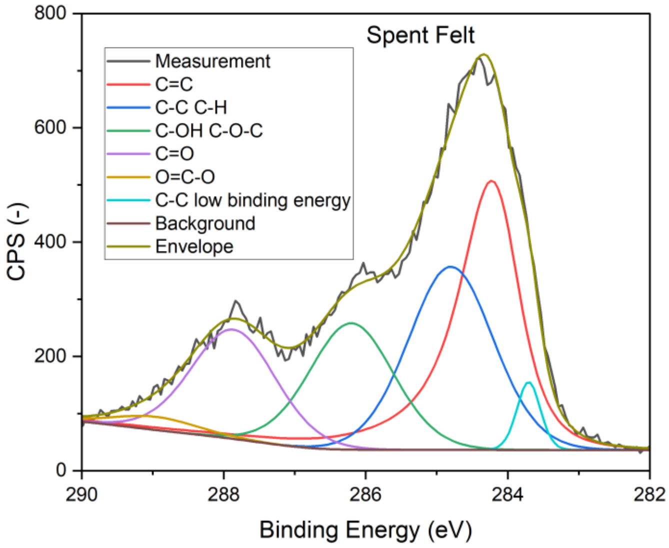

Fig. S11: XPS spectrum and fit of pristine carbon felt

References

[1] S. Claramunt, A. Varea, D. López-Díaz, M. M. Velázquez, A. Cornet, A. Cirera, *The Journal of Physical Chemistry C* **2015**, *119*, 10123–10129.
